# Broad Dataset and Methods for Counting and Localization of On-Ear Corn Kernels

**DOI:** 10.3389/frobt.2021.627009

**Published:** 2021-05-14

**Authors:** Jennifer Hobbs, Vachik Khachatryan, Barathwaj S. Anandan, Harutyun Hovhannisyan, David Wilson

**Affiliations:** ^1^Intelinair, Inc., Champaign, IL, United States; ^2^Intelinair, Inc., Yerevan, Armenia; ^3^The Robotics Institute, Carnegie Mellon University, Pittsburgh, PA, United States

**Keywords:** counting, density estimation, precision agriculture, dataset, YOLO, machine vision application, edge deployment, UNET

## Abstract

Crop monitoring and yield prediction are central to management decisions for farmers. One key task is counting the number of kernels on an ear of corn to estimate yield in a field. As ears of corn can easily have 400–900 kernels, manual counting is unrealistic; traditionally, growers have approximated the number of kernels on an ear of corn through a mixture of counting and estimation. With the success of deep learning, these human estimates can now be replaced with more accurate machine learning models, many of which are efficient enough to run on a mobile device. Although a conceptually simple task, the counting and localization of hundreds of instances in an image is challenging for many image detection algorithms which struggle when objects are small in size and large in number. We compare different detection-based frameworks, Faster R-CNN, YOLO, and density-estimation approaches for on-ear corn kernel counting and localization. In addition to the YOLOv5 model which is accurate and edge-deployable, our density-estimation approach produces high-quality results, is lightweight enough for edge deployment, and maintains its computational efficiency independent of the number of kernels in the image. Additionally, we seek to standardize and broaden this line of work through the release of a challenging dataset with high-quality, multi-class segmentation masks. This dataset firstly enables quantitative comparison of approaches within the kernel counting application space and secondly promotes further research in transfer learning and domain adaptation, large count segmentation methods, and edge deployment methods.

## 1. Introduction

Corn yield is driven both by optimizing the number of plants per area as well as the number of full, mature kernels on an ear. Disease, pests, weather, and nutritional challenges can cause ears to fail to develop properly, reducing the farmer's yield for that field. Depending on the variety of corn, each ear may have 400–900 kernels when fully developed; manually counting each kernel is slow, inaccurate, and labor intensive. Effectively automating such a process would provide the farmer with substantial speed and accuracy improvements. Developing such a model to aid farmers is the motivation for this work; recent advances in computer vision and machine learning enable the development of highly accurate models including those which may be deployed directly to a mobile device and run in the absence of high-speed internet.

The ultimate goal of this vision system is to enable farmers to make real-time management decisions using the most accurate information available. For kernel counting, this may involve a farmer in a field, taking a photo with a smart-device such as a phone or tablet, and receiving an accurate, real-time response with both count and localization information. Alternately, it may be embedded on robotic equipment which traverses the field for monitoring and management. Importantly, high-speed internet is often lacking in these scenarios and therefore having an edge-deployable solution is key. In addition to comparing several counting by detection approaches, we also demonstrate the utility of counting by density-estimation for such a task. Such approaches often have simpler architectures than detection models, which enables them to be more easily deployed on edge or mobile because they are smaller in size and lack sophisticated layers like ROI-pooling layers which can cause trouble in the conversion processes.

Corn kernel counting is not a new task for computational agriculture (Velesaca et al., 2020; Wu et al., 2020). However, past analyses have been done on proprietary datasets which prevents comparison across approaches. Very recently, Khaki et al. (2020) developed a CNN-based sliding-window approach as opposed to using SOTA detection methods like SSD, YOLO, or Faster R-CNN because “these methods need considerable amount of annotated images which do not exist publicly for the corn kernel detection.” This statement is a key motivator for creating this dataset; SOTA methods which have proven so valuable in the broader computer vision community should not be out of reach for precision agricultural because relevant datasets are kept private.

The objective of this work is thus 3-fold:

1. [Application] Baseline three deep learning approaches for counting and localization of on-earn kernels which can be deployed to a mobile device.
2. [Dataset] Release a dataset to enable development and comparisons of approaches for such an application.
3. [Dataset] Further construct this dataset to enable future research around high-count small-object detection and segmentation, domain adaptation, lightweight models for edge deployment, and other areas to advance precision agriculture.

Our belief is that with the release of this dataset and demonstration of the success of these three different deep-learning methods, agriculture applications will continue to move toward greater adoption of state-of-the-art deep learning methods which have been successful in so many other domains.

**Figure 1:**
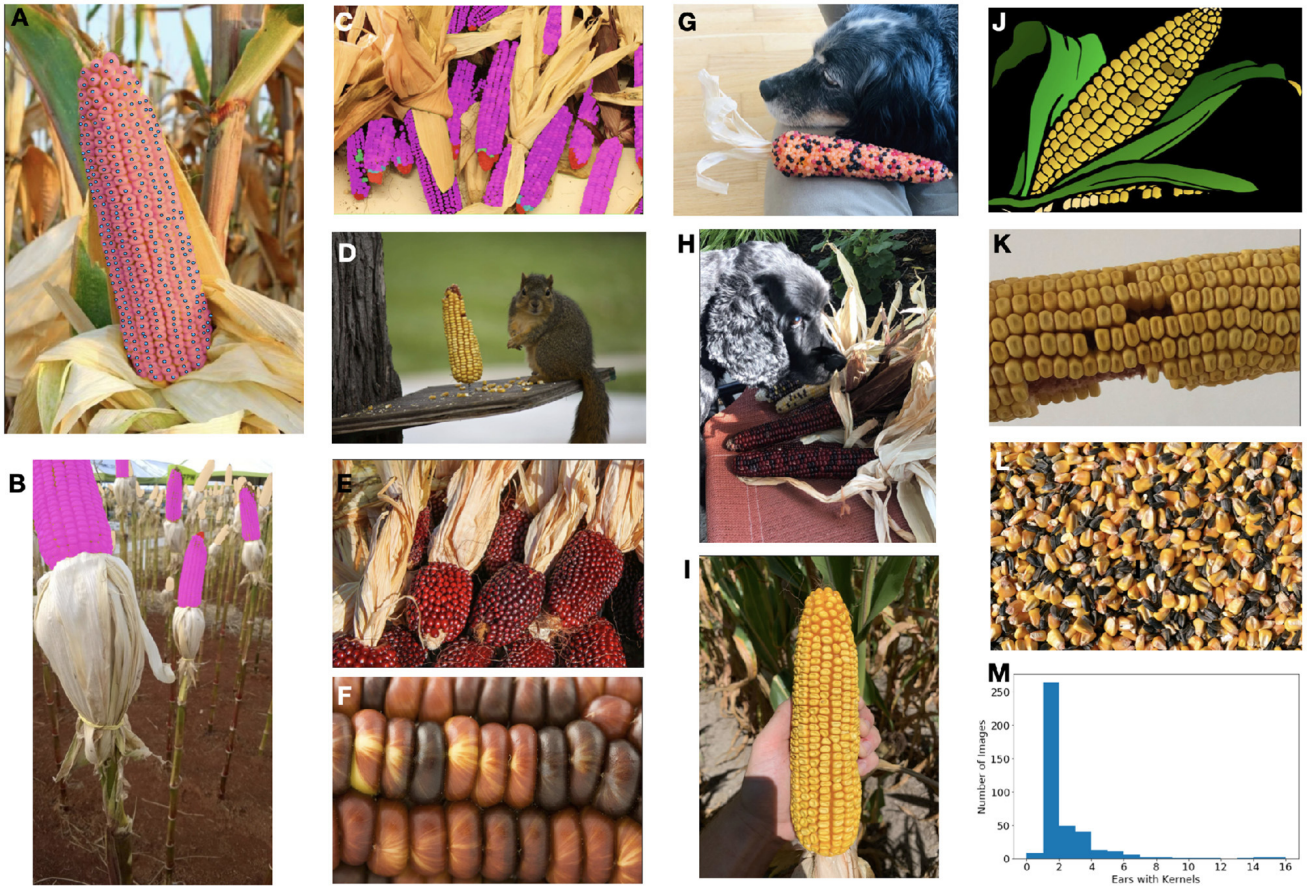
The ultimate goal of this model is count and localize the healthy kernels on ears of corn. **(A)** Our dataset is densely labeled with per-instance segmentation masks (area shown in faded magenta with centers in blue). **(B,C)** In addition to healthy (magenta) kernels, we also label barren tips (red), incomplete/underdeveloped kernels (cyan), diseased kernels (green), and kernel areas (beige). **(D–L)** The dataset contains a diverse range of images with different types of corn, numbers and sizes of ears and kernels, photos and cartoon-imagery, shadowing and occlusion challenges, scales and resolution, as well as kernels which are loose, and therefore should be ignored. **(M)** The distribution of the number of ears in each image in the full dataset.

By releasing this dataset (Figure 1), we seek to create a standard against which different approaches may be compared. Additionally, many past works are focused on a narrow range of data in which the corn variety, image size, resolution, background appearance, number of ears, or orientation of the ear are restricted. How a model's performance will transfer to an unseen dataset or generalize across datasets is therefore unknown. This is perfectly acceptable when the primary goal is to develop a model which supports a particular application; in that case, many of those sources of variation may be controlled for or ignored as out-of-scope for the given application.

However, as humans we are able to count and localize corn kernels trivially across a wide range of appearances and conditions; it is a decidedly “system 1” task that even young children can complete with ease (Kahneman, 2011; Booch et al., 2020). Thus from a machine learning perspective, we would desire that our intelligent system be capable of generalizing to broad domains as well. In addition to these theoretical considerations, the ability to learn from different data domains has key practical relevance; acquiring sufficient data from the desired domain is not always possible, especially when seasonal effects come into play. Instead, we would desire that our model learn from all available sources of data, with minimal effort required (here in the form of annotated data) when it is exposed to a new domain.

To address this, we constructed our dataset to consist of three sub-components. The *Base* portion of our dataset is designed to be “challenging,” with a wide range of corn varieties, diseases, lighting conditions, number of kernels visible, and broad appearances including synthetic images. We further supplemented the base component with a further challenging *Many* portion which contains images with many ears and very high numbers of kernels; such a task remains simple (albeit time consuming) for a human, but causes many SOTA computer vision models to fail. Finally, the *Narrow* portion of the dataset consists only of those images from the single application-specific domain (i.e., one corn variety, vertically oriented images, all of the same size). Formulating the dataset in such a manner will additionally enable future work in transfer learning and domain adaptation approaches.

Constructing the kernel counting application alone, per-image count, center-point, or bounding-box annotations of healthy kernels could have proven sufficient. To extend the applicability and breadth of this dataset, we also annotated diseased and incomplete kernels as well as barren tips and kernel areas (where kernels are present, but too indistinct to count) in addition to healthy kernels. For all classes, we have provided instance masks of these multiple classes to encourage the exploration of instance and panoptic segmentation methods that can handle hundreds of instances. The multi-class composition results in a highly imbalanced dataset, providing additional challenges and avenues of exploration.

## 2. Related Works

### 2.1. Kernel Counting

Kernel counting has been explored previously using traditional computer vision and image processing techniques as well as deep learning-based approaches. A distinct advantage of image processing-based techniques is that they do not require annotated data for model training. In 2014, Zhao et al. (2014) performed on-ear kernel counting using multiple preprocessing steps including Otsu thresholding (Xu et al., 2011), wallis filters (Mastin, 1985), and histogram enhancement; while performance was high, it was demonstrated on only 20 images of single ears of maize, taken on a black background.

Recently, while deep learning techniques have come to dominate much of the computer vision space, Grift et al. (2017), Li et al. (2019), and others continue to use image processing techniques; as their task of interest was highly constrained—often taken in a laboratory setting with a simplistic background, controlled lighting, loose kernels which naturally have separation —the flexibility and generalization offered by deep learning methods may not have warranted the effort of collecting a dataset to support deep learning methods. Even more recently, Wu et al. (2020) used a five-step approach consisting of Gaussian Pyramids, Mean Shift Filtering, Color Deconvolution, local adaptive thresholding, and local maxima finding to count the kernels on an ear of corn. While their results were good (> 93% accuracy reported) and conducted using two lighting conditions, the backgrounds were simplistic, all 8 maize varieties were yellow or white, and the approach requires 0.64 s to process a single image, quite slow compared to current SOTA deep learning-based approaches.

As deep learning approaches have shown superior performance across numerous machine vision tasks in multiple domains, they have been increasingly adopted for kernel counting tasks as well. Velesaca et al. (2020) used Mask R-CNN to segment individual corn kernels as well as to classify them as good, impure, or defective. This analysis was done on loose, not on-ear, kernels with a highly uniform background. Nevertheless, they demonstrated the resounding performance of their deep learning approach over traditional image processing (i.e., watershed) or hybrid (i.e., U-Net plus watershed) approaches. Very recently, Khaki et al. (2020), motivated by a lack of detection-enabling annotations (e.g., bounding boxes, instance masks, point annotations), used a CNN trained to classify individual kernels and then deployed as a sliding window across the entire image at inference.

### 2.2. Counting Overview

Our main task concerns counting the number of kernels on an ear of corn and therefore we draw heavily from the methods coming out of the crowd counting domain (Loy et al., 2013; Sindagi and Patel, 2018). These approaches tend to fall in one of three categories: counting by detection, counting by regression, and counting by density-estimation. If the sole goal is to obtain the count, counting by regression is a viable pathway. These methods seek to learn a set of either hand crafted or deep features, and then directly regress the total count without localizing the entities of interested (Chen et al., 2012, 2013). However, as the desired end application here provides the user with both the count and some validation or intuition behind that number (e.g., a point or bounding box around the entities to be counted), these approaches are less applicable here and therefore we focus on detection and density-estimation techniques.

#### 2.2.1. Counting by Detection

Counting by detection approaches leverage state-of-the-art detection models like Faster R-CNN (Ren et al., 2015), R-Fcn (Dai et al., 2016), RetinaNet (Lin et al., 2017), SSD (Liu et al., 2016), and YOLO (Redmon et al., 2016; Redmon and Farhadi, 2018) to detect, and subsequently count, the entities of interest in the image. Each of these approaches makes architectural decisions to balance speed vs. accuracy as detailed in Huang et al. (2017). In general, single-stage detectors like SSD and YOLO tend to be lighter weight and faster than two-stage detectors like Faster-RCNN, at the cost of accuracy, although performance is strongly dependent on the choice of backbone.

In addition to the framework, the choice of backbone also impacts performance and speed. Commonly use backbones include VGG (Simonyan and Zisserman, 2014), ResNet (He et al., 2016), EfficientNet (Tan and Le, 2019), and MobileNet (Howard et al., 2017), again with the speed/performance needs of the end application dictating the choice of architecture.

Recently, new detection paradigms have come to surpass the now “standard” approaches like Faster R-CNN. RetinaNet (Lin et al., 2017) leveraged focal loss and a dense detection model which performed at speeds comparable to one-stage detectors while outperforming two-stage detectors on the COCO benchmark. DETR (Zhu et al., 2020) used transformers with the same or fewer parameters to outperform Faster R-CNN on the COCO benchmarks. Similarly, EfficientDet (Tan et al., 2020) used a very lightweight bi-directional feature pyramid to drive enhanced performance. Although lightweight, these are not commonly deployed in mobile frameworks currently, and therefore not the focus of the present analysis.

Detection-based approaches work best in sparse counting scenarios where the entities are well separated, occlusions are limited, the number of entities is relatively low, and entities are larger in size (Elbishlawi et al., 2020). The computational efficiency of these models often scales with the number of detections and therefore can perform quite inefficiently in a dense-counting scenario (Arteta et al., 2016). Furthermore, the amount of memory required grows with the number of detections for processing the potential candidates; this can require a large amount of compute when the count is high, or require the image to be windowed if a high count is anticipated.

#### 2.2.2. Counting by Density-Estimation

In contrast to counting by detection, density-estimation approaches are tailored to scenarios where the number of entities may be quite large (potentially in the hundreds to thousands), occlusions and overlaps are present, and entities can be quite small in size (perhaps only a few pixels). These approaches tend to use architectures more characteristic of segmentation tasks such as the fully convolutional encoder-decoder structure of U-Net (Ronneberger et al., 2015). Recent work in this area has used more complex networks to handle the variations in size, scale, and perspective common in dense crowd counting scenarios (Boominathan et al., 2016; Sang et al., 2019; Xu et al., 2019). When localization is desired, but an exact bounding box is not required, density-estimation techniques provide a useful alternative to detection-based counting methods.

## 3. Methods

### 3.1. Dataset

We gathered 402images from publicly available data as well as privately acquired images. Images were annotated for (healthy) kernels, diseased kernels, “incomplete” (i.e., not fully matured) kernels, barren tips (i.e., where the top of the ear does not produce kernels), and “kernel areas” where on-ear kernels were present but resolution/lighting prevented annotation. After images were annotated, they underwent careful QA for needed changes and improvements. For the present work we only explore the healthy kernels; all other classes were ignored. The full dataset contains over 113,000 individual kernel segmentation masks and the associated bounding boxes in COCO format.

As seen in Figure 1, this dataset is highly varied in a number of manners including image size, resolution, number of ears present, total number of kernels present, background, lighting, corn variety, image type (i.e., photograph vs. cartoon), amount of zoom, etc. This is in stark contrast to the datasets used for many corn kernel counting applications which are restricted to the domain expected at inference: an outdoor image of a single ear of corn, belonging to a single (or limited set) corn variety, vertically oriented, at a roughly standardized size, at a standardized resolution, and with minimal shadowing or occlusions. Limiting the training domain in this manner can suffice if the domain at inference time is guaranteed to be similar; however, these models will likely not generalize well and may be unstable to unanticipated shifts in the inference domain. Instead, we elect to train the model on a broad set of data and then allow for fine-tunning to specific, narrower application-specific domains if needed.

**Figure 2:**
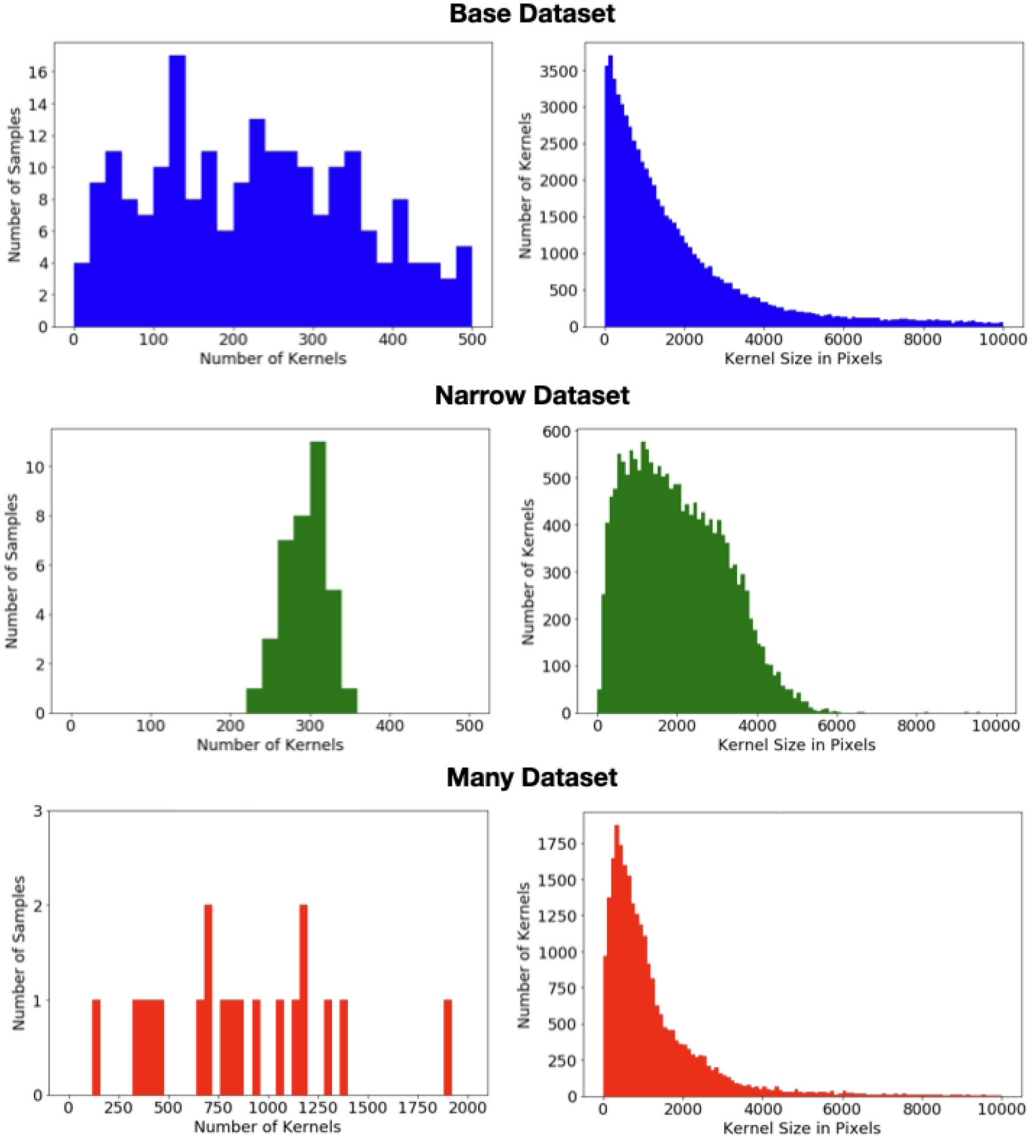
**(Left)** Distribution of the number of kernels per image in each of the three datasets. **(Right)** Distribution of the kernel size (i.e., area) in pixels in each of the three datasets.

To explore this impact, we divide the data into three datasets whose statistics are shown in Figure 2. The bulk of the data (313images) form the “base” dataset. These images have a wide range of appearances and may have multiple ears. A “narrow” dataset is compiled from 60we collected from taking photos of ears in several fields during the 2020 season. All of these images have a single ear of (solid) yellow feed corn, located roughly in the center of the image, vertically oriented, and with shadowing and occlusions largely minimized. Finally, while the base dataset contains several images with a very high kernel count, it is skewed toward simpler scenes. In contrast all 29images in the “many” dataset have at least 4 ears, a very large number of kernels, and often have on-ear kernels occupying the vast majority of the pixels. In the present work, we have not done significant exploration with this portion of the dataset as poses problems for both detection-based methods. However, we have chosen to include it in this release so that others may test their approaches on these very high-count examples.

### 3.2. Model Training

We divide each of three data subsets (base, narrow, many) into 60% train, 20% validation, and 20% test.

We used the albumentations (Buslaev et al., 2020) package to introduce significant augmentation during training on a per image, per epoch basis. Augmentations and their probabilities included: random rotation (*p* = 0.9), vertical flipping (*p* = 0.5), horizontal flipping (*p* = 0.5), random brightness contrast (*p* = 0.4), random gamma (*p* = 0.4), hue saturation (hue shift limit = 0.1, saturation shift limit = 0.1, value shift limit = 0.2, *p* = 0.5), random sun flare (*p* = 0.2), random shadow (*p* = 0.1), random rain (*p* = 0.2), Gaussian noise (*p* = 0.4, maximum = 0.01). All other parameters not noted here are the default parameters given in Buslaev et al. (2020).

Additional model-specific processing is described in the associated sections.

All models were constructed using the PyTorch framework (Paszke et al., 2019). Models were trained on a machine equipped with an Intel i7-10700 processor, 64GB of RAM, and two NVIDIA GeForce 1080Ti GPUs.

### 3.3. Metrics

For all models, we compute the Mean Absolute Percent Error (MAPE), also known as Mean Absolute Percent Deviation (MAPD), on the total count (which we refer to as “Count MAPE”) for the entire image. Count MAPE is given by

$$\begin{array}{l} \text{Count MAPE}=\frac{1}{J}\overset{J}{\underset{j=1}{\sum }}\frac{\left|Actual_{j}-Predicted_{j}\right|}{Actual_{j}} \end{array}$$

where *J* is the number of images, and Actual_*j*_ and Predicted_*j*_ are the actual and predicted number of healthy kernels in the image *j*, respectively. Note that for Faster R-CNN, the images must first be windowed to enable inference; Count MAPE is computed on the entire (recombined) image, not the windows individually.

For the detection models, we also compute the (mean) Absolute Precision at 0.5 intersection over union (IoU) written as “map@0.5” (Padilla et al., 2020).

$$\begin{array}{l} \text{IoU}=\frac{\text{area of overlap}}{\text{area of union}}\;\text{precision}=\frac{\text{true positives}}{\text{true positives + false positives}} \end{array}$$

mAP@0.5 is the mean absolute precision when the actual and predicted bounding box have at least 0.5 IoU.

### 3.4. Detection Methods

#### 3.4.1. Faster R-CNN

Faster R-CNN is a widely used detection model because of its accuracy; however its speed and size limits its usefulness for deployment on mobile devices. Although our end goal is to produce a model which can be deployed on mobile, we wish to benchmark that final model against the performance of a two-stage detection model like Faster R-CNN.

Therefore, we constructed two Faster R-CNN models with two different backbones: VGG-16 (Simonyan and Zisserman, 2014) and ResNet-50 (He et al., 2016). The choice of these two backbones was somewhat arbitrary: we sought two backbones with different sizes in this two-stage framework. However, alternative backbones including SqueezeNet (Iandola et al., 2016) or MobileNet (Howard et al., 2017) could be explored in the future. In both cases, the models were pretrained on Imagenet (Deng et al., 2009).

Out-of-memory errors can occur when the number of instances is large because each one must be held in memory and evaluated during processing. To counter-this, we split the training images into smaller windows for training and inference; images were split in half repeatedly until they were able to fit into memory; usually two divisions (a factor of 4) was sufficient. Alternatively, one can address such issues with larger machines with greater memory, however, as the focus of this work is on the creation of an application not on the optimization of training, we note this as a practical limitation of this approach. A goal of this work is to engage the broader community in this application, and therefore we solve this challenge “practically,” not with more compute, to make it more accessible.

We trained the model trained with a batch size of batch size of 1 using SGD with an learning rate of 0.005. All of these experiments are conducted with the *base* dataset. Training continued for up to 300 epochs unless halted early due to early stopping.

For Faster R-CNN we focused only on the base dataset because performance was seen to be noticeably worse than the other two frameworks in addition to being much more difficult to work with due to having to cut the image into smaller windows. As a result, we did not focus on analyzing the narrow dataset in the Faster R-CNN framework as it would be exceedingly difficult to use in the mobile application setting that the narrow dataset seeks to capture.

#### 3.4.2. YOLO

Since the original YOLO framework was introduced, there have been a number of improvements with subsequent versions. We chose to focus on the recent YOLOv5 (Jocher, 2020): although YOLOv5 has not been released with a formal publication, significant colloquial evidence has shown it has outperformed many other models, particularly for mobile deployment. Briefly, YOLOv5 is a single-stage detector with a model backbone, neck, and head. It uses a Cross Stage Partial Network (CSP) (Wang et al., 2019) as its backbone, PANet (Liu et al., 2018) as its neck, and the same model head as in YOLOv3 and YOLOv4 to generate output vectors with class probabilities, bounding boxes, and objectness scores. Unlike previous versions, it attempts to learn the anchor box locations using kmeans and a genetic algorithm. This architecture emphasizes LeakyReLU activation functions in the middle layers and sigmoid activation at the final output layer. We compared the impact of using weights pretrained on COCO data vs. beginning with randomly initialized weights.

We first train the model on the base dataset and calculate the desired loss and metrics. We also compute the Mean Absolute Percent Error on the total count for the unseen *narrow* dataset. We compare this result to training on the smaller narrow dataset directly. Each model was trained using SGD with a learning rate of 0.01, batch size of 12, for a maximum of 300 epochs.

### 3.5. Density-Estimation

#### 3.5.1. Density-Map Creation

Density-estimation approaches only require point annotations, unlike the previous methods which require bounding-boxes; to generate these point annotations we took the centroid of each instance-mask and placed a single point annotation at that location.

That is, we define a sample image *I* with *M* pixels ***x****m* and the associated set of *N* kernel annotations masks ***K*** = {***k***_1_, ***k***_2_, ..., ***k***_*N*_} where each ***k***_*n*_ = {***x***_1_, ***x***_2_, ..., ***x***_*P*_} is the set of 2D pixels in that individual kernel mask. The centroid of a given mask is then defined by $\bar{k_{n}}=\frac{1}{P}\overset{P}{\underset{p=1}{\sum }}x_{p}$ We convert this set of pixel masks to point annotations defined by $Z=\left\{z_{1},z_{2},...,z_{N}\right\}=\left\{\bar{k_{1}},\bar{k_{2}},...,\bar{k_{N}}\right\}$. Note that this approach does not require mask or bounding box annotations: directly annotating ***Z*** as point locations, would suffice.

Once all annotations for that image were converted to points, we applied an isotropic Gaussian blur with σ = 12 across the point-annotation mask to generate the target density map. That is we define the ground-truth density map ***D*** to be a kernel density estimate given by:

(1) $$\begin{array}{l} D\left(x_{m}\right)\overset{\text{def}}{=}\overset{N}{\underset{n=1}{\sum }}N\left(x_{m};z_{n},\sigma^{2}1_{2\times 2}\right) \end{array}$$

(2) $$\begin{array}{l} =\overset{N}{\underset{n=1}{\sum }}\frac{1}{\sqrt{2\pi \sigma }}\exp\left(-\frac{∥x_{m}-z_{n}{∥}_{2}^{2}}{2\sigma^{2}}\right) \end{array}$$

Because the Gaussian is infinite and therefore can extend well outside the original mask's boundaries, we truncated all values below 1*e*^−4^ and then renormalized the remaining occupancy mass so that it sums to 1 as required.

#### 3.5.2. Model Architecture and Training

All of the models used for the density estimation approach leverage a U-Net style architecture with different backbones: EfficientNet-b1 and MobileNet-v2 with ImageNet pretrained weights in the encoder. During training, the images were randomly cropped to 480 × 640 and at validation/testing, the image was split into windows of the same size. The choice to crop was not due to any memory limitations, but because we wished to expand the variability of the data seen during training.

We leverage the PyTorch Segmentations Models package for ease of analysis (Yakubovskiy, 2020). We used Adam Optimization with an initial learning rate of 0.0005, a batch size of 10, and a maximum of 300 epochs.

Finally, we define the loss for each of these models to be the pixel Mean Squared Error (pixel MSE) between the ground-truth and predicted density maps given by,

(3) $$\begin{array}{l} MSE=\frac{1}{M}\overset{M}{\underset{m=1}{\sum }}||D\left(x_{m}\right)-\hat{D}\left(x_{m}\right){||}_{2}^{2}. \end{array}$$

## 4. Results

### 4.1. Detection Models

Results for the Faster R-CNN and YOLOv5 models trained (and tested) on the base dataset are shown in Table 1. Note that Faster R-CNN and YOLOv5 use different loss functions so those should not be compared directly; instead the mAP@0.5 and mean absolute percent error (MAPE) is useful for comparing the performance of these models. In addition to not requiring pre-processing to split the image into smaller windows, the YOLOv5 model outperformed both Faster R-CNN models.

**Table 1:** Detection model performance.

	**Training data**	**Test loss**	**mAP@0.5**	**Count MAPE**	**Narrow count MAPE**
Faster R-CNN: ResNet50	Base	0.80	0.74	0.21	–
Faster R-CNN: VGG16	Base	0.85	0.73	0.24	–
YOLOv5: CSP	Base	0.70	0.70	0.24	0.20
YOLOv5: CSP, no pretraining	Base	0.74	0.64	0.25	0.29
YOLOv5: CSP	Narrow	0.63	0.63	0.17	0.17

Not surprisingly we see that using pretrained weights made a difference both on the in-domain dataset (test loss = 0.70, count MAPE 0.24) as well as the narrow dataset (test loss = 0.63, count MAPE 0.17), compared to using random weights (test loss = 0.74, count MAPE 0.25).

With the goal of creating a model which can count and localize kernels on a (presumably) single ear of corn, training and inferencing on this specific, narrow domain could be advantageous, provided enough data exists: the narrow dataset is an “easier” dataset relative to the base, as the images are much more stereotypic. However, gathering a dataset with sufficient sample in the target domain is not always feasible and therefore the ability to learn from the broader base dataset is also valuable. When trained and tested on the base dataset, the MAPE on the (test) base dataset is 0.24. When tested further on the (unseen, different domain) narrow dataset, the MAPE falls to 0.20. This captures the “difficulty” of the base dataset; the model performs better on the unseen narrow dataset than on the unseen test portion of the base dataset on which it was trained. However, when trained directly on the narrow dataset, the MAPE on the (test) narrow dataset is 0.17. (Note that “Count MAPE” and “Narrow Count MAPE” correspond to the same thing in this row). This does not mean that the broader base dataset is not useful; using the base dataset for initial training and then fine tuning on the narrow dataset is likely to be advantageous and a focus of ongoing work.

**Figure 3:**
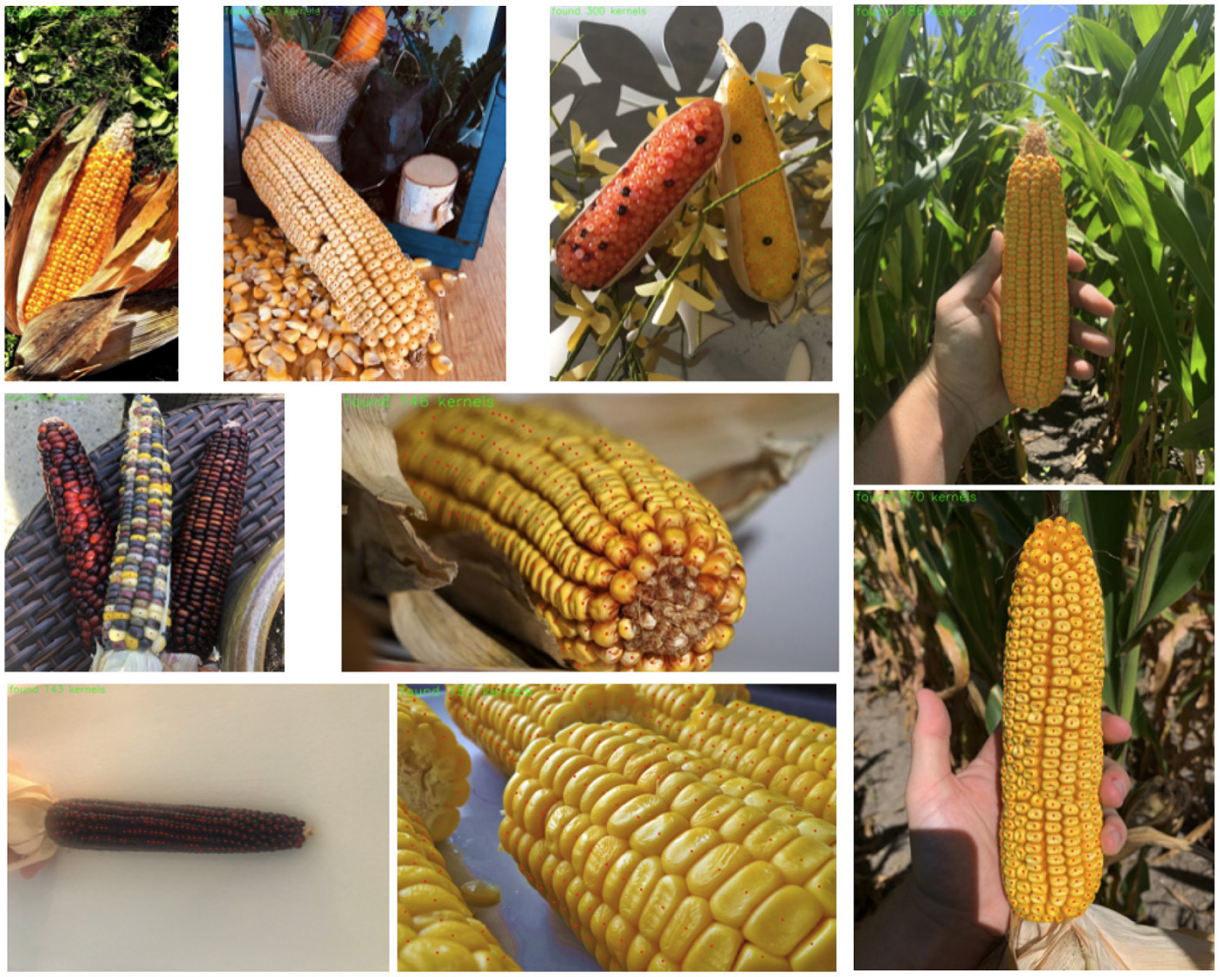
Results from YOLOv5 model trained on the base data. Samples from the base test set are shown on the left and middle and two samples from the unseen test narrow set are shown on the far right.

Results from the YOLOv5 model (pretrained weights) trained on the base dataset are shown Figure 3. Overall we see that these results are very impressive: the model is detecting the vast majority of the kernels, even on many “unusual” ears with different colors and kernel shapes, although it seems to struggle most with solid, dark-red kernels like the one seen in the bottom left. Impressively, the model has learned to ignore loose kernels which have detached from an ear. Furthermore, inference time for this model is 26 fps (0.038 s/image) on the GPU.

### 4.2. Density-Estimation

Results for the density estimation approach are seen in Table 2. EfficientNet-b1 and Mobilenet-v2 are both relatively small backbones with 6M and 2M parameters, respectively, and performed roughly equivalently across all tasks.

**Table 2:** Density estimation model performance.

	**Training data**	**Pixel MSE loss**	**Count MAPE**	**Narrow count MAPE**
EfficientNet-b1	Base	0.12	0.18	0.19
Mobilenet-v2	Base	0.18	0.19	0.18
EfficientNet-b1	Narrow	0.16	0.18	0.18
Mobilenet-v2	Narrow	0.16	0.15	0.15

**Figure 4:**
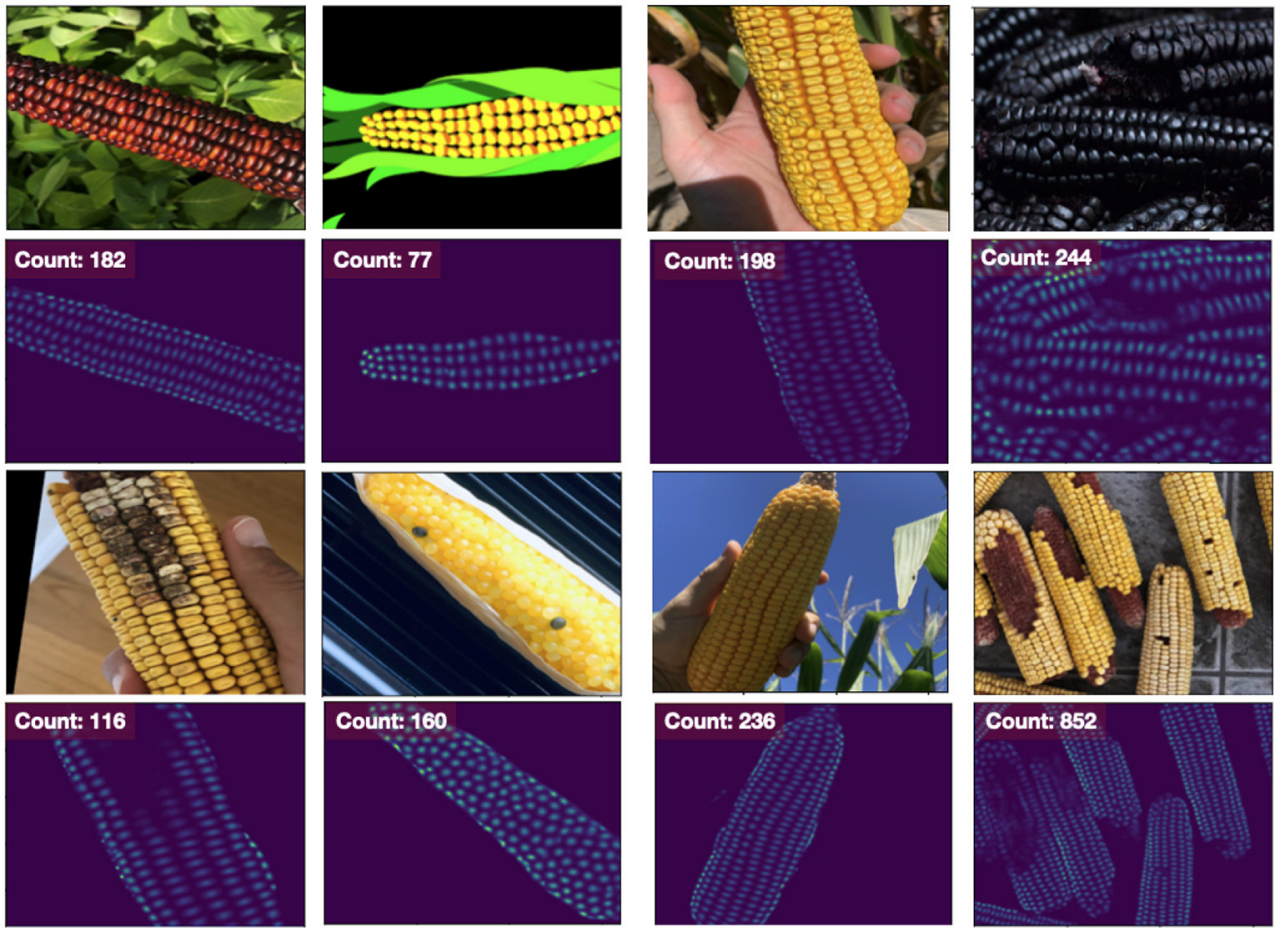
Results from our Density Estimation approach using an EfficientNet-b1 model. Other models are visually similar. Even though it was trained on the base dataset (left two columns), it performs quite well on the narrow (third column) and many (far right) dataset as well. Even though the number of instances may be large, the model does not struggle with memory issues and continues to be computationally efficient.

Furthermore, both density-estimation models outperform the detection-based approaches on the MAPE for both the base and narrow datasets. From the visualization of the output from the EfficientNet-b1 model trained on the nase dataset, we see that the model has learned to handle numerous challenging scenarios including different varieties, disease, missing kernels, cartoon images, difficult lighting conditions and shadows, and a large number of kernels in an image (Figure 4). While the “many” dataset is problematic for the detection methods, the density-estimation models handle these with ease (far right column of Figure 4); the Count MAPE on the unseen *many* dataset for this model is 0.23. While higher than the MAPE on the base or narrow subsets, this result is still quite impressive as these images contain very high amounts of occlusion and often extremely small instances.

While we have cropped the images for training to boost the variety of the dataset, there is no need to crop the data, as in the case of Faster R-CNN, or resize the image, as occurs naturally in YOLOv5. As those steps can dramatically slow the performance of the overall application, this is a key advantage for the density-estimation approach. The inference time for this model is 200 fps (0.005 s/image) on the GPU across all three data subsets (base, narrow, many).

## 5. Discussion

### 5.1. Model Performance

In the present work we have constructed a dataset and compared the performance of three different deep learning-based approaches for on-ear corn kernel counting and localization. The single-stage YOLOv5 model was found to outperform the two-stage Faster R-CNN approach in addition to being faster and more memory efficient, working directly off the raw images as opposed to requiring windowing. The U-Net based density estimation approach also performed quite well, matching or exceeding the Count MAPE and Narrow Count MAPE compared to the best YOLOv5 model. An additional advantage of the U-Net model is its ability to handle samples from the many datset, which remains problematic for the YOLOv5 model due to memory constraints. Qualitative comparison suggests that the U-Net is able to find the smallest kernels on the sides of the ear which are only slightly visible better than the YOLOv5 model; this is likely due to the fixed anchor resolution of YOLO models which is a known limiting factor.

Both the YOLOv5 and U-Net models met speed and accuracy requirements necessary to deploy to our mobile application and have their advantages and disadvantages in the application setting. While the output of the U-Net provides the localization required to provide the user confidence in the predicted count, it is more difficult for the user to make a “mental correction” if he notices that a kernel is missed and simply increment or decrement the provided count. We could enhance the U-Net's visual output by placing a peak-finding algorithm on top, but that would reduce computationally efficiency. Next, because the application is expected to see only a single, vertically oriented ear, some of advantages the U-Net holds in the general setting become less relevant within this particular application. Similarly, because all images in the application are of the same size, the fixed image-size (via padding or reshaping) that YOLO models require is not a concern. While the U-Net-based density estimation framework offers many advantages on the general modeling front, the YOLOv5 model was almost as performant on the narrow dataset and offers a more effective visualization to the user out of the box from an application viewpoint.

### 5.2. Future Work

The absence of sufficient annotated data has hindered the adoption of deep learning approaches in domains such as computational agriculture. The dataset created for this present work, while motivated by a single application, is highly complex and opens the door for many lines of research while still having real world significance.

First, we have explored only a few frameworks and backbones in the present work. While massive speed-accuracy benchmarking studies have been conducted in the past (Huang et al., 2017), those were done on large public datasets like COCO (Lin et al., 2014). Performing a similar large-scale analysis on this dataset which has a limited number of classes, but large number of entities per image could prove illuminating.

Additionally, this dataset supports studies around transfer learning and domain adaptation. We demonstrated that when trained on the base dataset, the models performed as well or better on the narrow, target domain. We would anticipate that by first training on the base dataset and leveraging domain adaptation techniques, this performance would only further increase. Additionally, it is interesting to ask whether given a target domain, is the entire base dataset equivalently useful for training? That is, is the broadest possible dataset the best for initial training, or are some samples too different and should be discarded or phased out during the training process?

Next, while the dataset was annotated with instance masks, we used only the associated bounding boxes (for the detection methods) or instance centroids (for the density-estimation methods). Performing instance or panoptic segmentation would be highly valuable, however, current SOTA methods do not scale well to hundreds of instances as required by this task. We hope this work and dataset motivate the development of segmentation methods which can handle such a large number of entities.

We have focused only on the healthy kernels for the present analysis as it is the central component of the motivating application. However, the dataset contains multi-class labels; whether learning the additional classes aids in the primary task is the focus of future research. Incorporating these additional classes introduces the challenge of handling significant class imbalance, as all of the other classes are extremely rare compared to the healthy kernels.

For researchers specifically interested in edge-deployment, this dataset and benchmarks offer a starting place to explore numerous topics in this area so performance can be compared. Since the outset of this work, newer models like EfficientDet and DETR have shown improved performance over the models explored here. However, they have not yet been exhaustively tested in the small-size-high-count realm like the present or made easily deployable to mobile through the support of necessary layers; we hope this work will encourage both. The development of other lightweight frameworks and backbones, quantization methods, as well as improved target hardware itself would all benefit from further study.

## 6. Conclusion

An edge-deployable automated kernel counting model is a key application for improving management decisions for farmers. Such a model may be used on a mobile device during inspection of a field or on robotic inspection and treatment devices which are becoming more prevalent in precision agriculture. In this work we have constructed several different models from the counting-by-detection and density-estimation paradigms with an eye toward fast, lightweight models which could be deployed to mobile. While we have by no means performed an exhaustive study on the different varieties and parameterizations of detection and density-estimation techniques, we have established that there are numerous ways to produce accurate, lightweight, deep learning-based counting and localization models, amenable to mobile deployment, for this key agricultural task.

Central to this work is the creation and release of a dataset which includes over 300 challenging images from a broad domain, 60 images of highly constrained images which mirror those likely seen by such a mobile application, and a smaller set of 29 images containing a large number of ears and kernels. A key goal of creating this dataset was to engage researchers on both the algorithm and application sides of machine vision for agriculture and we believe this dataset is a powerful step in this direction.

## Data Availability Statement

This dataset has been made availability on the AWS Registry of Open Data under the name Intelinair Corn Kernel Counting.

## Author Contributions

DW and JH conceived, planned, and oversaw the experiments. JH, VK, BA, and HH conducted the modeling and experiments. VK, BA, and JH collected data. JH led and supervised the annotations. JH led the manuscript writing with contributions from all authors. All authors contributed to the article and approved the submitted version.

## Conflict of Interest

All authors were employed by IntelinAir, Inc. during this work.
